# Mammalian Expression of Virus-Like Particles for Advanced Mimicry of Authentic Influenza Virus

**DOI:** 10.1371/journal.pone.0009784

**Published:** 2010-03-22

**Authors:** Chia-Ying Wu, Yi-Chun Yeh, Yu-Chih Yang, Ching Chou, Ming-Tsan Liu, Ho-Sheng Wu, Jia-Tsrong Chan, Pei-Wen Hsiao

**Affiliations:** 1 Agricultural Biotech Research Center, Academia Sinica, Taipei, Taiwan; 2 Center for Disease Control, Department of Health, Taipei, Taiwan; 3 Genomics Research Center, Academia Sinica, Taipei, Taiwan; Institute of Bioengineering and Nanotechnology, Singapore

## Abstract

**Background:**

Influenza A viruses are major human and animal pathogens with huge economic and societal impact from illness, hospitalizations, and deaths. Virus-like particles (VLPs) of influenza virus have been suggested as a vaccine candidate offering improved safety and efficacy. To develop this concept further, we established a flexible platform to efficiently generate different subtypes of mammalian-expressed influenza VLPs. Here we demonstrate that these mammalian VLPs strongly resemble the authentic viruses in structure, particle size and composition of host factors, and even glycosylation of viral antigens.

**Methodology/Principal Findings:**

In this study, a mammalian VLP system was established by stable co-expression of four influenza structural proteins (HA, NA, M1, and M2) in a Vero cell line. By replacing the surface glycoproteins of HA and NA, we converted the H3N2-VLP subtype to H5N1-VLP. After centrifugation purification of conditioned media, the particle morphologies, average sizes, and hemagglutination abilities of secreted VLPs were characterized, and the VLP constituents were identified by LC/MS/MS. Protease protection assays demonstrated that specific cellular proteins that co-purified with influenza virions were integrated into mammalian VLPs. The glycosylation profiles of mammalian VLPs as revealed by deglycosylation assays were similar to that of progeny viruses produced from Vero cells. Vaccination of mice with 2.5 µg and above of H5N1-VLP elicited H5-specific IgG1 antibodies and resulted in full protection against lethal infection with homologous virus. These results provide compelling evidence that mammalian VLPs closely emulate the exterior of authentic virus particles not only in antigen presentation but also in biological properties and should provide promising vaccine candidates.

**Conclusions/Significance:**

This flexible mammalian influenza VLP system offers a superior alternative to the conventional reverse genetic vaccine platform without concerns over inadequate presentation of immune antigens or limitations imposed by the manipulation of real viruses.

## Introduction

Influenza infection is a major threat to human health and results in significant morbidity and mortality worldwide. According to World Health Organization estimates, seasonal influenza epidemics influence 5∼15% of the global populations annually and are responsible for more than 3–5 million hospitalizations and about 250,000 to 500,000 deaths per year (http://www.who.int/mediacentre/factsheets/fs211/en/index.html). Recently, in addition to the yearly circulating seasonal influenza variants caused by antigenic drift, other influenza virus strains with pandemic potential such as the highly pathogenic avian H5N1 or emerged novel A/H1N1 pose greater threats than in the past (http://www.who.int/csr/disease/avian_influenza/country/en/ and http://www.who.int/csr/don/2009_08_19/en/index.html) since they have become better adapted to humans by reassortment. The most efficient way of reducing the transmission of and the subsequent huge economic loss caused by seasonal or pandemic outbreaks of influenza is preventive vaccination. The manufacture of the current licensed influenza vaccines, either in the form of a split subvirion (disrupted, highly purified virus) or a subunit vaccine (purified hemagglutinin, HA, and neuraminidase, NA), is absolutely dependent on fertilized chicken eggs as a production bioreactor. This method has substantial limitations since the manufacturing capacity is restricted by the availability of eggs, which may be insufficient to meet the urgent requirements for vaccine during a pandemic [1], [2]. In addition, these vaccines induce antibodies primarily to the viral HA and are efficacious in healthy adults, but display lower protective rates in high-risk groups (e.g., the elderly) and may be poorly immunogenic in young children. These problems are compounded once the wild population of virus undergoes significant antigenic drift in the HA component [1], [3], [4], [5], [6]. Consequently, the protective immunity elicited by inactivated vaccines is of too short a duration to protect from newly developed influenza variants. Therefore, the development of vaccines with cross-protective efficacy to allow a rapid response to influenza evolution and/or to prolong the efficacy of vaccination needs to be addressed.

Recently, the use of noninfectious virus-like particle (VLPs) that self-assemble by spontaneous interactions of viral structural proteins has been considered to offer good potential for advanced vaccines for a wide range of viruses that cause disease in humans [7]. It is worth noting that a VLP-based human papillomavirus (HPV) vaccine produced in yeast system which is capable of inducing protective immune response against the HPV responsible for cervical cancer was approved for the market in 2006 [8]. Influenza VLPs expressed by recombinant baculovirus systems that present multi-component antigens, including HA and matrix 1 (M1), with or without NA, and that are capable of inducing cognate or innate immune responses against homologous or heterologous strains of influenza virus, have been widely described [9], [10], [11], [12], [13], [14], [15]. Alternatively, a further improvement in the preparation of seasonal influenza vaccines licensed in Europe uses reverse genetics in mammalian cell-based culture systems rather than in eggs [6]. Using mammalian cell culture systems such as Vero or MDCK cells as adaptive hosts for vaccine viruses has several advantages, not only increasing the flexibility and consistency of the manufacture process but also recovering the host-dependent specific glycosylation of viral antigens which may not be glycosylated properly in egg- or baculovirus-dependent systems. In eukaryotic cells, protein glycosylation is involved in correct folding or directing the cellular localization of newly translated proteins and plays important roles in protein function. Different glycosylation patterns underlie some of the differences between various strains of the influenza virus. Generation of mammalian influenza VLPs has previously been achieved by transient coexpression of HA and NA proteins in human 293T cells [16]. For vaccine production purposes, we included here not only HA and NA but also the matrix proteins M1 and M2 in our mammalian VLP expression system since both M1 and M2 have equally critical roles in influenza virus assembly and budding processes, suggesting their similar importance for mammalian VLP budding efficiency [17], [18], [19], [20], [21], [22]. The incorporation of M1 and M2 into mammalian VLPs not only increased the VLP production yield but also supplemented interior viral antigens, which may provide highly conserved T-cell and B-cell epitopes to fight homologous and heterologous viruses [23], [24].

In light of the great threats posed by seasonal and pandemic influenza infection, any improved means for the development of effective and safe vaccine candidates is worthwhile pursuing. In this study, we have developed a flexible platform for the efficient production of mammalian influenza VLPs by simply co-expressing four influenza virus proteins, HA, NA, M1, and matrix 2 (M2) in Vero cells. We have successfully created mimics of two different subtypes of virus by replacement of surface antigens HA and NA. These noninfectious mammalian VLPs mimic the authentic virions not only in their morphology and functional HA but also other important constituents. The close similarity of the glycosylation patterns of the viral surface antigens (HA) seen between VLPs constructed by transformed Vero cells and authentic virus which had been expanded in Vero cells implies that these nonpathogenic mammalian VLPs have similar antigenicity to wild virus particles. Taken together, these findings suggest that this mammalian VLP system presents a practical new approach to safe and effective vaccine production, which has none of the drawbacks of the egg-based or the baculovirus culture-based methodology, and is an alternative to the conventional reverse genetics approaches used in influenza vaccine manufacture.

## Results

### Establishment of mammalian system generating influenza virus-like particles (VLPs)

**Figure 1:**
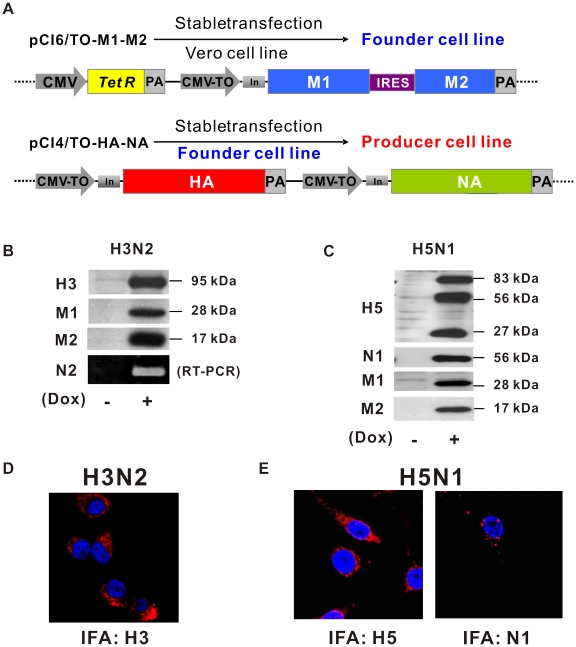
Construction and cellular expression of influenza virus-like particles (VLPs) in mammalian cells. (A) Diagrams of mammalian expression vectors of influenza VLPs. Indicated are the CMV constitutive promoter (CMV), tetracycline repressor gene (*TetR*), polyadenylation signals (PA), CMV doxycycline (Dox)-inducible promoter (CMV-TO), chimeric intron (In), encephalomyocarditis virus internal ribosomal entry site (IRES), and influenza genes (M1 and M2, matrix 1 and 2; HA, hemagglutinin; NA, neuraminidase). (B), (C) Expression of viral genes in selected quadruple VLP producer cells. The total cell lysates were extracted from VLP producer cells without (−) or with (+) Dox induction, and then probed with respective specific antibodies against HA, NA, M1, and M2 in Western blot assays. Expression of N2 was detected by RT-PCR. Molecular masses of expressed HA, NA, M1, and M2 are indicated on the right. (D), (E) *In vivo* immunofluorescence staining of expressed viral proteins in VLP producer cells with antibodies against HA and NA (red) as marked and counterstained with DAPI (blue).

In this study, we used mammalian cell culture-based approaches to generate influenza VLPs. To stably transfect the viral genes of HA, NA, M1, and M2 critical for VLP production into cultured Vero or Vero E6 cell lines, we designed four gene expression cassettes and placed these into two vectors as illustrated (Fig. 1A). A tetracycline repressor gene and *tet* operator-regulated gene expression cassette were also inserted in the plasmid expressing M1 and M2, giving the vector pCI6/TO-M1-M2. By stable transfection of the M1–M2 vector into Vero cells, we generated founder cells that would not express the M1–M2 transgene until doxycycline (Dox) induction. Another two *tet* operator-regulated, CMV promoter-driven expression cassettes were inserted separately into the other plasmid carrying HA and NA genes, giving the expression vector pCI4/TO-HA-NA. After stable transfection of pCI4/TO-HA-NA vector into an M1–M2 founder cell line, a mammalian-expressed VLP system was established.

To verify that co-expression of all four viral genes was indeed driven by the inducible promoter (CMV-TO), total cell lysates of quadruple-transfected Vero cell line of H3N2 were analyzed by Western blot with specific viral antibodies against H3, M1, and M2 (Fig. 1B). As there is no available antibody to N2, we used RT-PCR to confirm the expression of the N2 gene. The cellular localization of H3 was also observed by confocal laser scanning microscopy (Fig. 1D). The utility of this system as an alternative platform to reverse genetics for vaccine development was shown by the simple substitution of a separate plasmid carrying the HA and NA genes of H3N2 with those of H5N1 (Figs. 1C and E). The resulting quadruple H5N1-VLP cell line again co-expressed HA and NA, this time of the H5 and N1 varieties. So far, we have obtained two subtypes of quadruple Vero cell lines that generated the putative H3N2- and H5N1-VLPs, respectively.

### Morphology and antigen presentation of purified H3N2- and H5N1-VLPs

**Figure 2:**
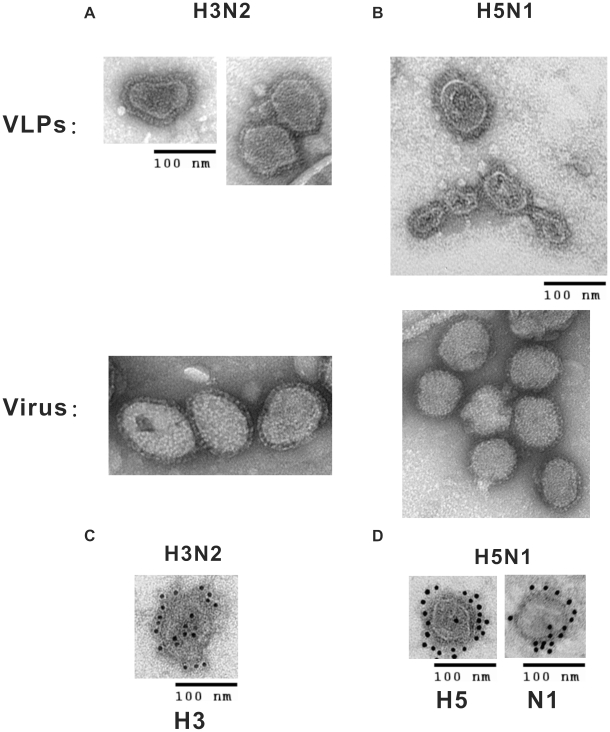
Morphology and antigen presence determinations of purified mammalian VLPs. (A), (B) Secreted VLPs and virus were purified by sucrose density gradient ultracentrifugation, negatively stained with 2% uranyl acetate, and observed by TEM at 100,000× magnification; pictures of respective virus strains are shown below the VLPs. (C), (D) Immunogold electron microscopy of purified VLPs. The primary antibodies used are shown below individual panels. Secondary antibody was goat anti-rabbit conjugated to 12 nm gold beads.

To investigate whether the H3N2- and H5N1-VLPs assembled and budded from the quadruple-transfectant cell lines, the media surrounding Dox-induced cells were collected and purified by cosedimentation in sucrose density gradient centrifugation. The morphologies of mammalian VLPs purified from culture medium of transfected cells were negative stained with 2% uranyl acetate and compared to their reciprocal viruses propagated in Vero cells using TEM (Figs. 2A and B). The VLPs displayed generally spherical morphologies and densely stained cores. The spike projections on the surface of VLPs were no different in appearance to those on authentic influenza viruses. The HA and NA glycoproteins on the surface of VLPs were immunogold labeled with individual specific antibodies and counterstained with gold spheres coupled to secondary antibodies (Figs. 2C and D).

### Dynamic light scattering (DLS) determination of average particle size of mammalian VLPs

**Figure 3:**
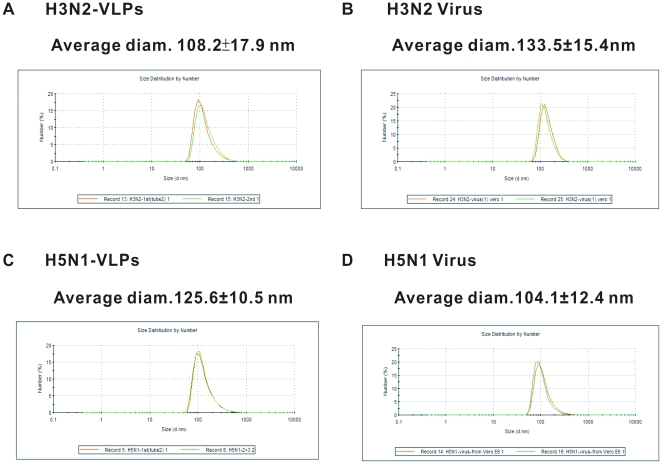
Dynamic light scattering (DLS) measurements of particle sizes and distributions of mammalian VLPs in solution. Average particle diameters of H3N2-VLPs (A), H3N2 virus (B), and H5N1-VLPs (C), and H5N1 virus (D) in phosphate buffer (pH 7.4), at 25°C. Two representative determinations of different batches of VLPs and virus are shown as red and green lines. The size distribution of VLP populations ranged from 70 to 200 nm with a 95% confidence interval (CI).

To complement the morphology analysis by TEM, the average sizes of secreted VLPs in native solution were estimated by DLS assays. To be an effective vaccine, it has been proposed that particles ranging from 20 to 200 nm could facilitate the drainage of free antigens to the lymph nodes and induce strong responses in dendritic cells (DC) for long-term protective purposes [25]. Laser-based DLS can monitor changes in Brownian motion of nanoparticles in solution, giving information related to the average size and frequency distribution of particles. As shown in Fig 3, the average diameters of H3N2- and H5N1-VLPs, as determined by DLS, were 108.2±17.9 nm and 125.6±10.5 nm, respectively (at pH 7.4, 25°C), and are comparable to the sizes obtained for their reciprocal viruses (H3N2: 133.5±15.4 nm and H5N1: 104.1±12.4 nm). The size distributions of both subtypes of VLPs ranged from 70–200 nm (95% CI), suggesting that the mammalian VLPs were in the preferred size range for DC uptake and promise to stimulate a potent immune response (Fig. 3). DLS will be a useful approach to monitor the batch-to-batch consistency of VLPs by rapidly providing information on the whole population of particles. Together, the DLS and TEM measurements of mammalian VLPs were consistent and show the VLPs to be of comparable size and morphology to native influenza viruses.

### Identification of VLP composition and VLP-associated cellular proteins

**Figure 4:**
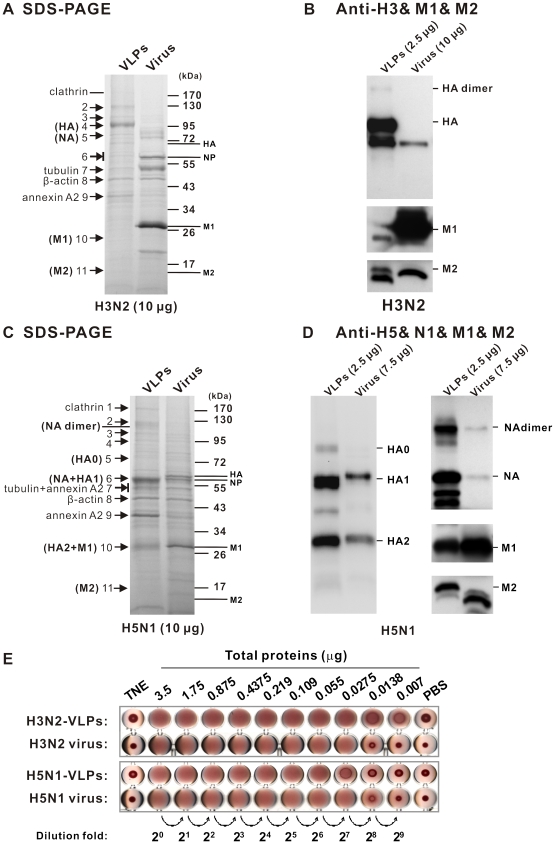
Characterization of purified mammalian VLPs. The total proteins of purified H3N2-VLPs and H3N2 virus (A) and H5N1-VLPs and H5N1 virus (C) were resolved by SDS-PAGE in a 7.5–17.5% gradient gel and stained with Coomassie blue. Molecular masses of protein markers are shown on the right. Each relevant band, as marked with an arrow and a number, was subjected to LC/MS/MS analysis to identify its composition. The identified viral protein bands of H3N2- and H5N1-VLPs (marked with parentheses in panels A and C) confirmed by Western blot analyses with relevant specific antibodies for H3N2-VLPs and virus (B) and for H5N1-VLPs and virus (D). The relative abundances of HA and NA, quantified by Chemigenius 2 (SYNGENE, Frederick, MD) and GeneTools (version 3.07) software, were 4∶1 for H3N2-VLPs and 3∶2 for H5N1-VLPs. The HA protein attributes to 12.8% and 18% of total proteins in H3N2- and H5N1-VLPs, respectively. (E) Assessment of HA function by hemagglutination assay. The amounts of mammalian VLPs or virus used are indicated, in a twofold serial dilution. TNE (buffer of VLPs) and PBS were included as the negative controls in the hemagglutination assays.

To verify the protein constituents of mammalian-expressed VLPs, 10 µg of H3N2- and H5N1-VLPs were separated on a 7.5–17.5% gradient gel, and stained with Coomassie blue (Fig. 4A and C) or probed with specific antibodies against viral proteins in separate experiments (Fig. 4B and D). Besides the viral proteins HA, NA, M1, and M2, we also observed a wide spectrum of minor bands in the VLPs similar to the authentic viruses (Fig. 4A and C). To identify the basic protein profiles of these VLPs, the more evident protein bands (indicated by arrows in Fig. 4A and C) were excised from the gels, subjected to in-gel trypsin digestions, and analyzed by liquid chromatography-tandem mass spectrometry (LC/MS/MS). The major VLP constituents identified by searching against the NCBI database are shown as Tables 1 and 2. We found that HA and NA proteins of mammalian VLPs were distributed in multiple gel slices in addition to the expected locations shown in Table 1. This likely reflects the fact that they are the most abundant proteins in VLPs and form HA and NA protein oligomers (Fig. 4B and D). In addition, we identified another 22 VLP-associated cellular proteins that are identical or functionally analogous to those cellular proteins commonly found in the interior or exterior of influenza virions [26]. Most of them could be classified into functional groups including cytoskeleton protein, extra cellular matrix (ECM) proteins, heat shock proteins, annexins, tetraspanins, and glycolytic enzymes. It is worth noting that we also identified several unique cellular proteins (listed in Table 2) with very high Mascot scores, which are possibly involved in VLPs biosynthesis. For real viruses, recruitment or encapsidation of some cellular proteins into the virion may be a critical behavior supporting the completion of the life cycle by some specific interaction with viral proteins or RNA. However, in this case, the mammalian influenza VLP has the components of viral (by transfection) and cellular proteins (by recruitment) without package of any viral genetic material. The cellular proteins identified in our VLPs might be actively involved in the normal virus life cycle, especially during virus assembly and budding from the host cells.

**Table 1:** Mammalian VLPs associated viral and cellular proteins identified by mass spectrometry (LC/MS/MS).

SDS-PAGE and LC/MS/MS Analysis						
Protein Type	Protein Name	Mass (Da)	Protein band number^a^H5N1/H3N2*	Mascot score^b^	Sequence coverage (%)^c^	Reported in influenza virion^d^
Viral proteins	Hemagglutinin (H5)	64163	5, 6, 10$	227, 812, 1053	26, 18, 40	yes
	Neuraminidase (N1)	51298	6	71	10	
	Hemagglutinin (H3)	63595	/4	/239	/24	
	Neuraminidase (N2)	52018	/5	/230	/40	
	Matrix 1 protein (M1)	27860	10/10	35/45	36/16	
	Matrix 2 protein (M2)	11157	11/11	34/44	50/35	
Cytoskeletal proteins	β-actin	41710	8/8	1519/447	72/73	yes
	β-tubulin	49639	7/7	41/655	15/72	yes
	myosin IA	118204	2/2	175/41	7/2	tropomyosin 4 & 1
	Similar to myosin IC	107102	3/3	898/107	42/15	
ECM proteins	integrin alpha 3	118333	2/2	896/69	27/20	Integrin beta 1
	integrin alpha 5	115919	2/2	687/174	37/22	
Heat shock proteins	Heat shock 90kDa protein	83185	4/4	435/368	40/43	HSP 27 kDa
	Heat shock 70kDa protein	72288	5/5	615/503	54/50	
	Heat shock 27kDa protein	22768	6	112	44	yes
Annexin	annexin A11	54443	9/7	284/282	38/38	yes
	annexin A2	53564/38576	7, 9/7, 9	1087, 361/153, 613	43, 41/26, 49	yes
Tetraspanin	CD81 molecule	25741	6	117	33	yes
	CD9 molecule	25380	7	27	11	yes
Glycolytic enzymes	enolase 1	47182	9	1011	70	yes
	Similar to phosphoglycerate kinase 1	44558	8	572	71	phospho-glycerate kinase
	Pyruvate kinase	64479	1/6	248/354	45/51	yes
	glyceraldehyde-3-phosphate dehydrogenase	35959	6/6	64/48	23/25	yes
Unclassified proteins	2′,3′-cyclic nucleotide 3′ phosphodiesterase	47509	8	306	56	yes
	aldo-keto reductase family 1	35992	7	99	23	yes
	WD repeat domain 18	48167	8	78	18	WD repeat-containing protein 1
	gamma-glutamyltransferase 1	61261	6/6	76/71	4/10	yes
	peroxiredoxin 2	21878	7	28	9	peroxiredoxin 1

a Excised protein bands were numbered as indicated by arrows 1–11 of Fig. 4A and C.

b For this search a Mascot score ≥25 is significant (p>0.05).

c Sequence coverage is based on peptides with unique sequence.

d Viral and cellular proteins have been reported in influenza virion [26].

*The band number, Mascot score, and sequence coverage of cellular proteins both identified in H5N1- and H3N2-VLPs were signalized as A/B.

$ The band number, Mascot score, and sequence coverage of single protein distributed in multiple locations was presented as A, B.

**Table 2:** Unique cellular proteins identified by LC/MS/MS with high Mascot scores in mammalian VLPs.

Protein Name	Mass (Da)	Protein band number^a^H5N1/H3N2*	Mascot score^b^	Sequence coverage (%)^c^
clathrin heavy chain 1 (Cytoplasmic vesicles formation	192276	1	3242	63
Spectrin (interact with actin)Beta (non-erythrocytic)	274472	1	1146	35
Plexin B2 (interact with cytoskeleton)	203451	1	757	26
Smilar to CD109	161515	1	738	23
Prostaglandin F2 receptor negative regulator	116885	2/2	1035/136	44/18
Na+/K+-ATPase alpha 1	112838	3/3	1266/452	42/29
Tumor rejection antigen (gp96) 1	92555	3/3	1016/614	52/60
Flotillin I	47384	9	1048	68

a Excised protein bands were numbered as indicated by arrows 1–11 of Fig. 4A and C.

b For this search a Mascot score ≥25 is significant (p>0.05).

c Sequence coverage is based on peptides with unique sequence.

*The band number, Mascot score, and sequence coverage of cellular proteins both identified in H5N1- and H3N2-VLPs were signalized as A/B.

To characterize the functionality of the HA spike on mammalian VLPs, hemagglutination assays were performed (Fig. 4E). VLP preparations reacted with 0.75% guinea pig erythrocytes had significant hemagglutination activities, with titers of 2^7^ for H3N2-VLPs and 2^6^ for H5N1-VLPs in samples containing 3.5 µg of VLPs. Both H3N2 and H5N1 viruses of same amount as VLPs were at similar titers (2^7^) of HA activity. This result suggests that the HA spikes compassing around the surface of mammalian VLPs are in a native orientation and function as those of active authentic viruses.

### Confirmation of cellular proteins associated and incorporated into mammalian VLPs

**Figure 5:**
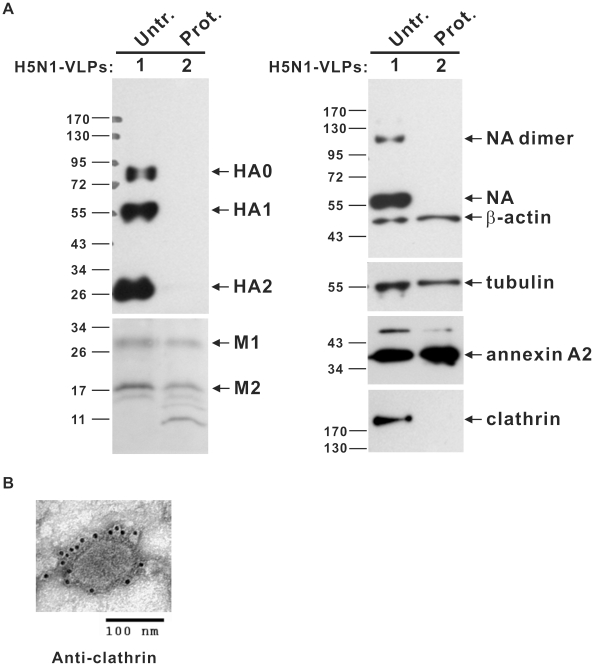
Specific integration of cellular proteins in influenza VLPs. Purified H5N1-VLPs were either mock-treated (Untr.) or digested overnight with typsin (Prot.) followed by purification with a 20% sucrose cushion. Mock-treated VLPs (lane 1) or typsin-treated VLPs (lane 2), 2.5 µg each, were further analyzed by Western blotting with antibodies against the indicated proteins including HA, NA, M1, M2, β-actin, tubulin, annexin A2, and clathrin (A). Molecular weight markers are marked on the left. (B) Immunogold labeling of clathrin on the surface of purified VLPs. Secreted VLPs purified from conditioned medium were immunogold labeled with antibody against clathrin, negatively stained with 2% uranyl acetate, and observed by electron microscopy (100,000× magnification).

Following proteomic identification of incorporated cellular proteins, several viral and associated cellular proteins were further characterized by Western blot and immunogold labeling. To rule out the possibility that identified proteins may be due to non-specific contamination such as co-purification of microvesicles or exosomes with VLPs, the mammalian influenza VLP preparations were subjected to a protease protection assay that has been shown to efficiently remove microvesicles from HIV-1 virion preparations [27], [28]. After trypsin incubation, H5N1-VLPs were re-purified by sucrose cushion ultracentrifugation and subjected to Western blot analysis with antibodies against HA, NA, M1, M2, β-actin, tubulin, annexin A2, and clathrin (Fig. 5A). Both HA and NA proteins were lost from the VLPs after trypsin digestion, demonstrating that these proteins were located on the outside of the membrane envelope, integral and attached to the VLP surface. Most co-purified contaminants were eliminated by the protease digestion. However, several representative cellular proteins expected to be inside the virion such as actin, tubulin, and annexin A2 were still found to be present in the protease-digested VLPs, which indicates that these cellular proteins were specifically incorporated into the structure of VLPs (Fig. 5A). In contrast, a unique cellular protein identified in this study, clathrin heavy chain, an important component of the clathrin-coated pits mediating the endocytosis of many receptors, ion channels, transporters, and other transmembrane proteins as well as various soluble macromolecules and viruses, was lost following protease treatment (Fig. 5A) [29], [30]. This finding raised two possibilities: Firstly, clathrin is associated with contaminants rather than the VLPs, or secondly, clathrin is indeed incorporated into VLPs but is exposed on the surface like HA and NA. To resolve this issue, we used immunogold labeling to look for the presence of clathrin on the surface of intact, undigested VLPs (Fig. 5B). This assay did show clathrin staining on the surface of intact VLPs, just as for HA and NA (Figs. 2C and D).

Generally, the results of two independent LC/MS/MS analyses of H5N1- and H3N2-VLPs and Western blotting in this study resemble the reported proteome of influenza virus [26], which suggests that the assembly of influenza VLPs shares much similarity with real virus assembly and escape. Taken together, these data confirm that the mammalian-expressed influenza VLPs are very similar to the authentic viruses, a considerable advantage to their use in further vaccine development.

### Glycosylation profiling of influenza VLPs

**Figure 6:**
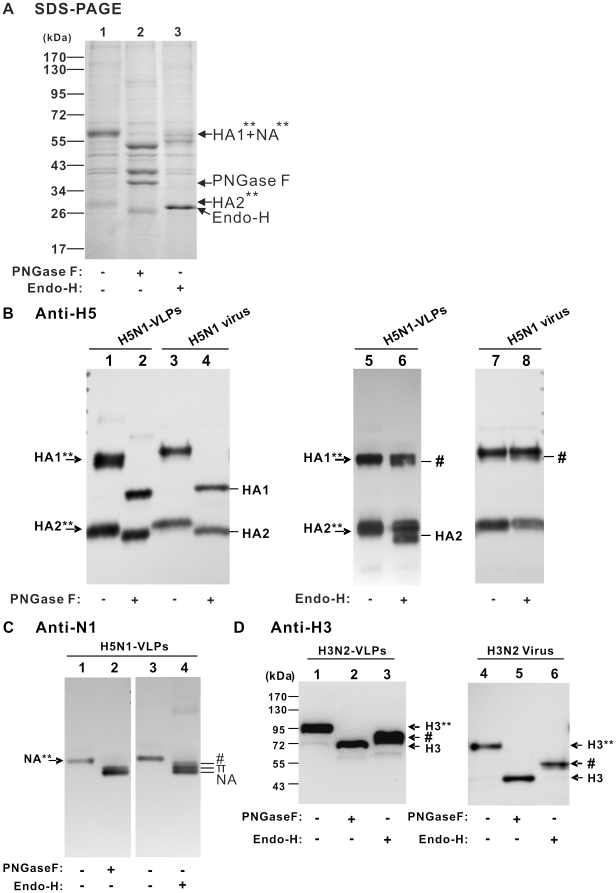
Glycosylation profiling of HA and NA in mammalian VLPs. Mock-treated and deglycosylase-treated proteins of H5N1-VLPs were separated by SDS-PAGE, stained with Coomassie blue (A), and further analyzed by Western blotting using antibodies against H5 (B) and N1 (C). In parallel, mock-treated and deglycosylase-treated proteins of H3N2-VLPs were analyzed by Western blotting using the antibody against H3 (D). Untreated and deglycosylase-treated proteins of respective VLPs and virus are compared in panels B and D. Glycosylated HA1, HA2, NA, and H3 are denoted as HA1**, HA2**, NA**, and H3**, respectively. **#** represents glycosylated proteins harboring a residue moiety of complex-type glycans sensitive to PNGase F, but not Endo-H. **π** represents unknown posttranslational modifications on the NA of H5N1-VLPs.

Glycosylation of viral surface antigens is critical for immune recognition, receptor binding, inflammation, and pathogenicity, and therefore has a major influence on the efficacy of vaccine antigens [31], [32]. For example, the common phenomenon of amino acid substitutions of the viral HA due to egg-adaptation and the consequent altered glycosylations severely affect the antigenicity of influenza virus [33]. As demonstrated by N-glycan footprinting analyses of HA, the use of different cell lines for replication of the same virus results in different N-glycosylation patterns on HA, which can be attributed to host-mediated changes in the amino acid sequence and potential glycosylation sites of HA, further influencing the antigenic properties of manufactured virus [34], [35], [36]. Therefore, the glycosylation status of HA and NA antigens in the VLPs were assessed to look for any change that would affect the antigenicity and immune response of a VLP-based vaccine. We compared the N-glycosylation patterns of H3N2- and H5N1-VLPs produced from Vero cells to the glycan profiles of authentic viruses by performing deglycosylation assays with N-endoglycosidases PNGase F and Endo-H. PNGase F can remove all types of N-linked oligosaccharides from glycoproteins such as complex, hybrid, and high-mannose types, whereas Endo-H cleaves the chitobiose core of high-mannose and hybrid oligosaccharides from N-linked glycoproteins. As shown in Figure 6A, most of the modified HA and NA (labeled as HA1**+NA** and HA2**) in purified H5N1-VLP was seen as two major bands in the SDS-PAGE gel before degylcosylation (lane 1); their apparent molecular masses were around 56 and 30 kDa, respectively (Fig. 6B and C, lanes 1). After treatment of H5N1-VLPs with PNGase F, HA1, HA2, and NA bands increased their mobility to molecular masses of 40, 27, and 52 kDa, respectively (Fig. 6A, B, and C, lanes 2), demonstrating that the two predominant viral surface antigens were mainly glycosylated by N-linked oligosaccharides. Of note, one form of NA marked as (π) in Fig. 6C whose mobility was not changed by reaction with both enzymes suggests NA may have other types of post-translational modification. When the glycosylated HA from H5N1-VLP was treated with Endo-H, the deglycosylation reaction was only partial; therefore the original bands and the Endo-H digested residue bands marked as (#) of HA1 and HA2 can be seen simultaneously (Fig. 6B, lane 6). However, the HA of H5N1 virus propagated in Vero cells was resistant to Endo-H digestion, suggesting the glycans linked to the viral HA are complex type (Fig. 6B, lane 8). The partial sensitivity of HA in H5N1-VLPs to Endo-H may be a result of hybrid glycan chains as the overwhelming expression of viral protein may lead to incomplete glycosylation. However, as the great majority of NA can be deglycosylated by Endo-H, the NA proteins of H5N1-VLP may possess more high-mannose than complex glycans (Fig. 6C, lane 4). In parallel, the deglycosylation assays were performed on H3N2-VLPs (Fig. 6D). With H3 glycoprotein, incubation with Endo-H or PNGase F reduced the molecular mass by 16 kDa or by 25 kDa, respectively, suggesting a higher content of high-mannose or hybrid types than complex type in the H3 glycan pool (Fig. 6D, lanes 2–3). Our results suggest that VLPs generated from this platform will have similar glycosylation profiles to the authentic viruses that result from infection in the same host species. Collectively, the mammalian VLPs resemble the real viruses in particle size, morphology, protein composition, and glycosylation profiles and therefore offer great potential as safe and effective influenza vaccine antigens.

### Humoral immune response of VLPs

**Figure 7:**
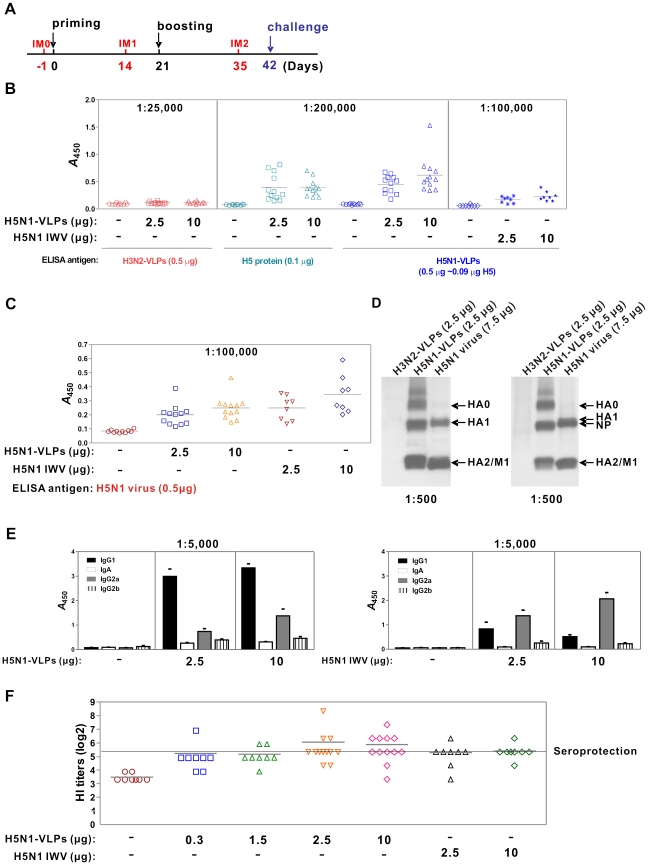
Humoral immune response of mammalian VLPs. (A) Regimen of prime and boost vaccination was followed by viral challenge. IM0, IM1, and IM2 represent the mouse serum collected before immunization (day -1), and 14 days after priming, and boosting, respectively. (B) The antigen-specific IgG antibodies from the serum of each mouse group taken at IM2 were assayed against distinct antigens of H3N2-VLP, H5 protein (recombinant baculovirus expressed), and H5N1-VLP (same as the immunization antigen) by ELISA after vaccination of H5N1-VLP or inactivated whole virus (IWV). Groups of mice (n = 8–12) were either intramuscularly immunized with 2.5 µg and 10 µg dose of VLPs, or IWV, as marked. The dilution of used serum samples in the ELISA assays are labeled at the top. H3N2-VLPs containing all the host cell proteins integrated into the VLP antigen but with different subtype of HA and NA was used as negative control, whereas the baculovirus-produced H5 protein constituted the positive control. The employed amounts of coating antigens are shown. (C) ELISA of serum IgG antibodies induced by H5N1-VLPs or IWV vaccines against H5N1 virus as ELISA antigen. (D) Western blot analysis of mice serum IgG antibodies elicited by either H5N1-VLP (left panel) or IWV (right panel) vaccines. The used antigens and individual amounts are indicated at the top of panels. (E) The specific IgG isotype and IgA elicited by VLPs and IWV vaccines were assayed using H5N1-VLP as ELISA antigen. (F) HI titer of each vaccinated mouse; mean values of the same group are plotted. HI titer of 40 was set as threshold of seroprotection.

To investigate the vaccine effect of mammalian expressed VLPs, we vaccinated mice with VLPs without adjuvantation and analyzed the antibody response and protection from viral infection. Mice (BALB*/c*; n = 12) were vaccinated twice (day 0 and 21) via intramuscular injection with purified H5N1-VLP or inactivated whole virus of H5N1-pseudotyped vaccine strain (recombinant H5N1 engineered by reverse genetics) at two antigen doses (2.5 µg and 10 µg). Blood samples were collected to analyze humoral immune response before primary (day -1) and after immunizations (day 14 and 35) (Fig. 7A). Sera were tested for influenza virus-specific IgG antibodies by ELISA against baculovirus produced H5 glycoprotein, or mammalian expressed H5N1-VLP and H3N2-VLP (Fig. 7B). Mice vaccinated with H5N1-VLP showed a robust response of IgG antibodies against H5 protein and H5N1-VLP. The ELISA titers of both antigen doses against H5 protein and H5N1-VLP were higher than 1∶200,000. By contrast, their titers against H3N2-VLP were insignificant (<1∶25,000). This suggests that H5N1-VLP was highly immunogenic, stimulating highly specific antibodies against the H5 epitopes. Whole virus vaccine resulted in ELISA titers of approximately 1∶100,000; while being highly immunogenic, the H5-specific titer was considerably lower than with the VLP vaccine (Fig. 7B). However, ELISA titers for VLP vaccine group reduced to the level of whole virus vaccine group when whole virus was used as ELISA antigen (Fig. 7C). This suggests that the immunity of H5N1-VLP was more potent and specific to H5 glycoprotein, which could stem from the higher HA content in the VLPs as compared to the one in the viruses. The specificity of the VLP-induced antibody was further accessed by Western blotting against all proteins of VLP and the virus. Indeed, the IgG antibodies detected only signals corresponding to the H5 glycoprotein and M1 in the VLP and virus (Fig. 7D, *left*). The lack of signal detecting other host proteins in this experiment indicated the VLP was as “clean” as the inactivated split virus and subunit vaccines, and predominantly immunogenic toward the HA glycoprotein.

The IgG antibody isotypes distribution elicited by vaccination is indicative of the type of T cell immune response, as subsets of antigen-specific helper T cells regulate the production of different IgG isotypes via secreting different cytokines. The IgG1 isotype in mice is believed to signal a Th2 response, whereas the IgG2a isotype indicates more of a Th1 response. ELISA test was further used to measure the class and IgG isotypes of antigen-specific antibodies in response to the VLP and whole virus vaccines. As shown, the antibodies induced by VLP were predominantly IgG1 isotype, much less in IgG2a, and low or undetectable in IgG2b and IgA (Fig. 7E, *left*). By contrast, the antibodies induced by whole virus vaccine were mainly IgG2a, less in IgG1, and negligible in IgG2b and IgA. These results suggest that mammalian VLP vaccine at the two antigen doses induced primarily a Th2 response, whereas whole virus vaccine stimulated a mixed Th1/Th2 response with Th1 more dominant at higher antigen dosage.

### Vaccine induced hemagglutination-inhibition (HI) activity and protection against viral infection

The HI assay is the most widely accepted serological test for influenza immunity and is the gold standard measure of functional HA-specific antibodies after vaccination. The serological criteria currently used for approval of pandemic vaccines in the US are based on seasonal influenza vaccines, with seroconversion (i.e. a minimum 4-fold rise in HI titer) rate >40% and seroprotection (i.e. HI titer >1∶40) rate ≥70% in adults younger than 65. Antibodies elicited by each vaccine candidate were evaluated for their ability to inhibit the VLP-induced agglutination of guinea pig red blood cells (Fig. 7E). After the second dose (day 35), the seroprotective HI titers were induced in 83.3% of mice that received 2.5µg and 10 µg VLP vaccine with mean HI titer reaching about 1∶60 (Fig. 7F). When the antigen dose of VLP vaccine was decreased to 0.3 µg and 1.5 µg, the reciprocal seroprotective rate dropped to 12.5% and 25%. Seroprotective rates of corresponding whole virus vaccine were 75% and 87.5%, respectively, in analogous experiments.

**Figure 8:**
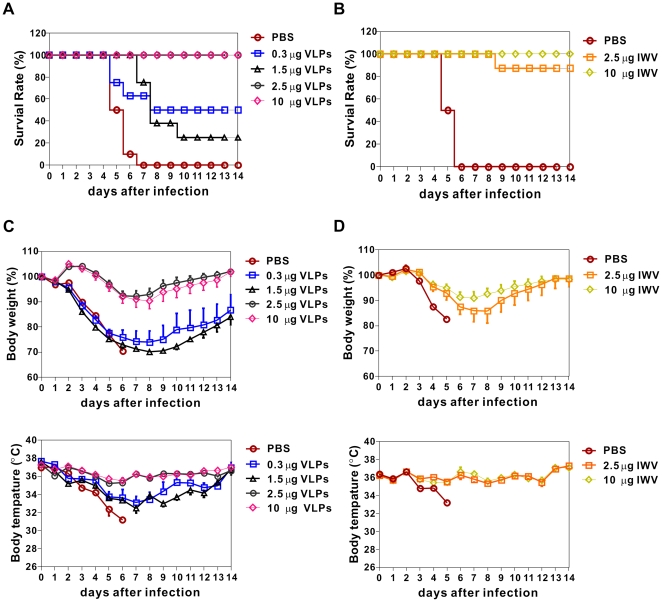
Vaccine Protection against lethal-dose challenge of H5N1 virus. At day 42, vaccinated mice were challenged intranasally with a lethal dose (100 LD_50_) of recombinant H5N1 (NIBRG-14) virus and monitored daily for weight loss and mortality. The percentages of survival rate and changes of body weight and temperature were recorded. (A) Survival for H5N1-VLP groups, (B) survival for IWV groups, Mice that lost greater than 30% body weight were euthanized. (C) Body weight and temperature for H5N1-VLP groups. For th groups receiving 0.3 µg and 1.5 µg antigen dose, only data of surviving mice are shown. (D) Body weight and temperature for IWV groups.

All mice vaccinated with VLP vaccine, whole virus vaccine or mock control were challenged intranasally with a predetermined lethal dose of H5N1-pseudotyped recombinant virus to evaluate the protective efficacy of each vaccine candidate. All mice which received 2.5 µg and 10 µg VLP vaccine survived after the viral challenge, in contrast to the mock control mice that all died within 7 days after infection (Fig. 8A). Lower dose (0.3 µg and 1.5 µg) of VLP vaccine compromised the survival rate (50% and 25%, respectively), which is consistent with a lower seroprotection rate. Whole virus vaccine (2.5 µg and 10 µg) resulted in protection against the viral challenge; the exception was one mouse in the 2.5 µg dose group that lost >30% body weight (Fig. 8B). Body weight and temperature changes of test mice were indicative illness and mice vaccinated with high-dose VLP and whole virus vaccine returned to their original weight by day 13 post-challenge (Fig. 8C, D), a result consistent with the survival outcome. However, mice vaccinated with low-dose VLP had more prominent weight loss and temperature decrease; nevertheless some of the survivors recovered at later time.

## Discussion

In this study, we have established a platform to generate mammalian influenza VLPs by the directed expression of four viral structural proteins (HA, NA, M1, and M2). We have also demonstrated the flexibility of this approach by exchanging the surface antigens of HA and NA to generate VLPs mimicking two subtypes of influenza (Figs. 2 and 4). When examined by TEM, the VLPs were found to closely resemble influenza virus in size, particle morphology, and fine structure of the surface spikes (Figs. 2 and 3). LC/MS/MS analyses and protease protection assays revealed that the cellular constituents specifically present in authentic virus were also incorporated into the mammalian VLPs, which implied that the particular interactions between host proteins and viral proteins involved in the biosynthesis of VLPs probably reflected similar processes during virus assembly and budding as the real virus (Table 1 and Figs. 4, 5). The glycosylation profiles of HA and NA spike in mammalian VLPs were examined by N-deglycosylation (Fig. 6). In both cases, the glycosylation profiles of HA were highly similar to that of influenza virus replicating in the Vero cells. Taken together, all these data demonstrate that VLPs produced by this mammalian cell-based system are a promising vaccine candidate.

Recently, clinical trials of HPV VLPs have led to FDA approval of the vaccine [37], [38]. Furthermore, influenza VLPs produced in baculovirus systems have shown promise as safe and high-yielding vaccines against influenza infection in preclinical animal models [9], [10], [11], [12], [13], [14], [15], [39]. Clinical studies for baculovirus-expressed influenza VLPs are currently being undertaken. In this study, we demonstrate that vaccination with mammalian expressed VLPs provided full protection against lethal infection with the homologous strain at doses as low as 2.5 µg VLP (0.45 µg HA) using two dose regimen in BALB*/c* mice. All these successes indicate that the VLP-based vaccine approach is an attractive alternative to replace or complement conventional inactivated virus vaccines or subunit vaccines with improved safety and efficacy, especially for children and the elderly.

In this study, we successfully applied a mammalian system to produce influenza VLPs on a preclinical scale by stable co-expression of four viral proteins. Typically, our pilot production in a 3 L-scale microcarrier system yielded an average 1.2 mg/L medium of mammalian VLPs after purification. Each inoculation of 3 L culture attained 10^9^ cells and could be induced to continuously express VLPs three times. This is also the first report to reveal, as confirmed by SDS-PAGE analysis, the proteome of mammalian VLPs with sufficient quantities of HA and NA proteins, including their further characterization by deglycosylation, hemagglutination assays, and immunization response.

Our proteomic analysis and protease protection assays of the secreted VLPs revealed that 22 cellular proteins associated with authentic virus of H1N1 were specifically incorporated into mammalian H3N2 and H5N1 VLPs (Table1 and Fig 5). Among these proteins, tubulin, actin, annexins, enolase, GAPDH, gamma-glutamyltransferase, and HSP 27 have been demonstrated to be derived from lipid raft by proteomic analysis in previous studies [40], [41], [42], [43], [44]. Correct assembly and budding of influenza virus requires cooperative action by multiple viral proteins with the lipid bilayers and genomic RNA as well as host proteins [45], [46]. In VLP systems, except for the interactions involved in viral RNAs and capsid proteins, the events related to virus particle release are thought to be highly similar to real virus assembly and release. A number of the proteins identified in mammalian influenza VLPs shed light on their roles in the influenza virion during assembly/budding stages of the infection process. The resemblance between VLP proteome and virus cellular proteins also suggests that the budding behaviors and constituents of mammalian VLPs are very similar to those of authentic viruses. Thus, VLPs expressed from a mammalian cell-based system constitute a non-pathogenic pseudovirion with virus-like properties and hold great promise as a vaccine candidate.

Apart from those common proteins identified in virion and VLPs, we found several unique VLPs-associated cellular proteins with very high Mascot scores in LC/MS/MS analysis. For instance, clathrin was specifically incorporated by binding to the exterior membrane of VLPs (Fig. 5). So far, it remains unclear why clathrin, an endocytosis mediator protein, was specifically associated with secretory influenza VLPs, though this discovery may have implications for the route of VLP biosynthesis and for the late stage of virus assembly and budding. Further experiments need to be designed for an explanation of this role.

The present study demonstrates, for the first time, the efficacy of VLPs created by a mammalian expression system as a new influenza vaccine. The full protection of mammalian VLP vaccine correlated well with functional antibody responses (HI assay), which is the licensure criterion accepted for yearly interpandemic vaccines. The presence of numerous cellular proteins integrated into mammalian expressed VLP may raise concerns of autoimmunity. Both Vero and Vero E6 cell lines are currently considered as the most widely acceptable cell substrate by regulatory authorities to produce a wide range of viruses for human vaccine production, including influenza, polio virus, rabies virus, smallpox, vesicular stomatitis virus, herpes simplex virus and rotavirus. Currently, the WHO recommends only the use of Vero cells to prepare viruses for vaccine production by reverse genetics [47]. For this reason, we chose Vero and Vero E6 cell lines rather than any other human or non-human mammalian cell lines to produce VLPs. In fact, our studies demonstrate that vaccination in mice with VLP without adjuvant formulation elicited high-titer antibodies only against HA but not other proteins (Fig. 7). Vaccinated mice survived perfectly with two doses of VLP vaccines at 2.5 µg and 10 µg levels via intramuscular immunization, and they all survived the subsequent lethal viral challenge. No adverse effects were found before and after viral challenge throughout the 56-day experiment. We should note that the humoral immune response elicited by mammalian expressed VLPs is different from that of baculovirus-derived VLPs, suggesting a distinction between the two forms of VLPs. Such difference may be attributed to the glycosylation profile, host protein contents, the overall particle structure that present antigens, or other factors.

In addition, this report focused on the characterization of advanced mammalian VLPs and the methodology of their production. We nonetheless propose that this will be a promising new agent to address influenza infection because 1) we have demonstrated here the preclinical-scale production of mammalian VLPs; 2) we have shown, by a simple exchange of surface antigens, that our development platform is flexible and can shorten the lead time for adjusting the match of vaccine specificity against the circulating strains of viruses; 3) this approach for the expression of VLPs will alleviate safety restrictions and bottlenecks associated with dependence on live viruses; and 4) this methology will permit rapid and scaleable production, independent of a reliance on egg availability for vaccine manufacturing.

## Materials and Methods

### Cells, plasmids, and antibodies

Vero cells were obtained from the Bioresource Collection and Research Center, (Hsinchu, Taiwan) and maintained in minimal essential medium (HyClone, South Logan, UT) supplemented with 10% fetal bovine serum (Gibco, San Diego, CA) in a humidified incubator at 37°C with 5% CO_2_. The cDNAs of M1 and M2 derived from the sequences of influenza A/Taiwan/083/2006 virus were cloned into the backbone modified from pcDNA6/TR (Invitrogen, Carlsbad, CA) linked by IRES separately into a single eukaryotic expression vector to give the plasmid pCI6/TO-M1-M2 (Fig. 1A). The cDNAs of HA and NA were synthesized sequences based on the distinct virus strains of A/Taiwan/083/2006 and A/Hanoi/30408/2005(H5N1) (a kind gift from Dr. Po-Huang Liang at the Institute of Biological Chemistry, Academia Sinica) optimized for mammalian codon usage and further cloned into expression vectors as illustrated for pCI4/TO-HA-NA (Fig. 1A). For the mammalian H3N2- and H5N1-VLPs producer cells, the plasmid pCI6/TO-M1-M2 was stably transfected into Vero cells to derive a founder cell line, which was further transfected with HA-NA expression vectors to obtain the quadruple co-expression cell line with HA, NA, M1, and M2 proteins. To confirm the gene expression of N2 in the H3N2-VLP producer cell line, total RNAs were extracted separately from cells with and without doxycycline (Dox) induction and RT-PCR assays were performed using a primer pair corresponding to the internal sequence of the N2 gene. These primers were N2-F, 5′-ATTAGGCT TTCCGCTGGTGGGGACAT-3′ and N2-R, 5′-GCATTCTGACTCCTGGGTCCTGA GGATT-3′. Expression of the proteins was confirmed by Western blot analyses and immunofluorescence staining as follows. Quadruple VLP-expression cells were induced with Dox for 48 h, or left untreated as a control. The cells were then fixed in 4% paraformaldehyde for 10 min and immersed in 0.05% Triton-X 100 for 1 min. After blocking with 1% gelatin, the cells were incubated with distinct primary specific antibodies, followed by incubation with goat anti-mouse or goat anti-rabbit IgG conjugated with Cy3 dye. Fluorescence images were acquired by confocal microscopy (LSM 510 META NLO DuoScan, Carl Zeiss, GmbH). The antibodies used in this study were polyclonal: H3 (ab20084), N1 (ab21305), M1 (ab20734), annexin A2 (ab41803), and clathrin (ab21679) from Abcam (Cambridge, MA), β-actin (sc-1616-R) from Santa Cruz Biotechnology (Santa Cruz, CA), and monoclonal: M2 (ab5416), and tubulin (ab6160) from Abcam and H5 (MCA2660, used for IFA) from AbD Serotec (Raleigh, NC). Rabbit polyclonal antibody against H5 used for Western blotting was provided by Dr. Che Ma (Genomics Research Center, Academia Sinica).

### Microcarrier culture and purification of mammalian VLPs

To scale up the cultivation of VLP producer cells, 60 g microcarriers (HyClone) and cells (about 2×10^8^) were added to a 3 L spinner flask (BellcoGlass, Vineland, NJ), stirred at around 35 rpm with a pendant glass ball, and maintained in minimal essential medium supplemented with 10% fetal bovine serum in a humidified incubator at 37°C with 5% CO_2_. After 7 days cultivation, the cells had attached to the surface of the collagen-coated microcarrier and grown to confluence. For VLP expression and secretion from cells, the culture medium was removed and replaced with serum-free medium (SFM4MegaVir, HyClone) containing 1 µg/mL Dox to begin induction.

After Dox-induction for 72 h, conditioned medium of VLPs producer cells was harvested, filtered with 0.45 µm Stericap, concentrated by Vivaflow 50 (Sartorius Stedim Biotech, Göttingen, Germany), and then layered onto a 30% sucrose-TNE (10 mM Tris-HCl, pH 7.4, 100 mM NaCl, 1 mM EDTA) cushion. Following centrifugation at 112,600×g for 2 h at 4°C in a Beckman SW28 rotor (Beckman Coulter, Fullerton, CA), the resulting pellet was resuspended in TNE buffer, and further purified over a 20–30–60% sucrose gradient (112,600×g, 2 h at 4°C). Finally, the banded VLPs were collected, dialyzed with TNE buffer overnight, and stored at −80°C. To analyze the protein constituents of purified VLPs, the samples quantified by Quant-iT Protein Assay Kit (Invitrogen) were mixed with Lämmle SDS-PAGE sample buffer, boiled for 5 min, and separated in a 7.5–17.5% gradient gel.

### Immunogold electron microscopy of purified mammalian VLPs

Sucrose gradient purified VLPs of 1 µg were adsorbed onto formvar/carbon-coated nickel grids (Electron Microscopy Sciences, Fort Washington, PA). After a 2 min wash with TBS buffer (50 mM Tris-HCl, pH 7.5, 150 mM NaCl), the sample was blocked with 1% BSA in TBS for 1 h. Primary antibody (10 µg/mL) was diluted in 1% BSA/TBS and adsorbed onto the grid for 1 h at room temperature. Following three washes with TBS, secondary gold-conjugated antibody was added for 1 h at room temperature. The grids were then washed twice with TBS, fixed with 1% glutaraldehyde, washed with water, and negatively stained with 2% uranyl acetate for 30 sec. The images of stained mammalian VLPs were captured using a Hitachi H-7000 transmission electron microscope.

### Dynamic light scattering (DLS) determination of particle size

Stock solutions of mammalian VLPs were diluted to 0.1 µg/mL in 20 mM phosphate buffer at pH 7.4, passed through 0.45-µm filters, and analyzed on a Nano ZS particle-size analyzer (Malvern Zetasizer, Malvern Instruments Ltd, UK). For each sample analyzed by DLS, two consecutive measurements were taken on a single sample and measured with a light-scattering data collection time of 60 s. The accompanying software (Nanov510) was used to convert the intensity-based measurement to a size distribution based on the number of particles in each size class, and was presented as a diagram of curves showing the frequency distribution of the sample where the area under the curve was proportional to the numbers of VLPs or virus detected in the relevant size range. The average diameters of VLPs or virus were then calculated as the mean size of particle population ± standard deviation (SD) of three independent experiments.

### Protease treatment of mammalian VLPs

Purified H5N1-VLPs equivalent to 50 µg of proteins were incubated for 18 h at 37°C with or without 20 µg of MSG-Trypsin (G-Biosciences, St. Louis, MO) in buffer containing 20 mM Tris-HCl (pH 8.0) and 1 mM CaCl_2_. The treated and untreated VLPs preparations were separately diluted to 7 mL with TNE buffer containing 30 µM PMSF (Sigma, St. Louis, MO), concentrated through a 20% sucrose cushion by ultracentrifugation (200,000×*g*, 2 h at 4°C in a Beckman SW41Ti rotor) and then subjected to Western blot analyses.

### Deglycosylation of mammalian VLP proteins

Purified VLPs equivalent to 10 µg of proteins were denatured by heating for 10 min at 100°C in the presence of 0.5% SDS and 40 mM DTT. Next, either PNGase F or Endo-H (New England Biolabs, Ipswich, MA) was added and the mixture incubated for 1 h at 37°C in 1×G7 or G5 reaction buffers (New England Biolabs). Subsequently, samples were subjected to protein gel electrophoresis and Western blot analyses.

### Identification of VLP relevant protein bands by LC/MS/MS

Proteins separated in one-dimensional polyacrylamide gels were sequentially isolated and subjected to in gel tryptic digestion prior to mass spectrometric analysis. Briefly, the protein bands from the 1-D gel were manually excised from the gel and cut into small pieces (∼0.5 mm^3^). The gel pieces were washed in a microcentrifuge tube with a solution containing 50% methanol and 5% acetic acid for 2–3 h, twice with a solution of 25 mM NH_4_HCO_3_ in 50% acetonitrile for 10 min each, and then dried in a vacuum centrifuge. After DTT reduction and iodoacetamide alkylation, a solution containing 75 ng of sequencing grade modified trypsin (Promega Corporation, Madison, WI) in 25 µL of 25 mM NH_4_HCO_3_ was added and incubated with dried gel pieces for 12–16 h at 37°C. Following digestion, tryptic peptides were extracted twice for 15 min each with 50% acetonitrile containing 5% formic acid under vortexing. The extracted solutions were pooled and evaporated to dryness under vacuum. The dried pellet was re-dissolved in 10–20 µL of 0.1% formic acid for LC/MS/MS analysis as described below. The NanoLC−nanoESi-MS/MS analysis was performed on a nanoAcquity system (Waters, Milford, MA) connected to an LTQ-Orbitrap XL hybrid mass spectrometer (Thermo Electron, Bremen, Germany) equipped with a PicoView nanospray interface (New Objective, Woburn, MA). Peptide mixtures were loaded onto a 75 µm×25 cm BEH C18 column (Waters) packed with 1.7 µm particles with a pore size of 130 Å and were separated using a segmented gradient from 5% to 50% solvent B (acetonitrile with 0.1% formic acid) over 90 min at a flow rate of 300 nL/min and a column temperature of 35°C. Solvent A was 0.1% formic acid in water. The mass spectrometer was operated in the data-dependent mode. Briefly, survey full-scan MS spectra were acquired in the orbitrap (*m/z* 350–1600) with the resolution set to 60,000 at *m/z* 400 and automatic gain control (AGC) target at 106. The 10 most intense ions were sequentially isolated for CID MS/MS fragmentation and detection in the linear ion trap (AGC target at 7000) with previously selected ions dynamically excluded for 90 s. Ions with single and/or unrecognized charge state were also excluded. The MS and MS/MS raw data were processed with Bioworks 3.3.1 and searched against an in-house generated NCBI protein database using Mascot Daemon 2.2 server. Search criteria used were trypsin digestion, variable modifications set as carbamidomethyl (C) and oxidation (M), allowing up to 2 missed cleavages, mass accuracy of 10 ppm on the parent ion and 0.60 Da on the fragment ions.

### Virus propagation, hemagglutination, and serological tests

Influenza virus A/Taiwan/083/2006 and H5N1 (NIBRG-14) strains (National Institute for Biological Standards and Control, Potters Bar, U.K.) were propagated in Vero (for VLP comparison) or MDCK cells (for viral challenge). To assess hemagglutination, 3.5 µg of VLPs or virus and their serial 2-fold dilutions were mixed with a 0.75% suspension of guinea pig red blood cells in 96 well plates. Plates were incubated for 1 h and hemagglutination was assessed by eye. The highest dilution of VLPs or virus giving hemagglutination was determined as 1 HA unit.

To assess hemagglutination inhibition (HI) titers, sera were treated with a receptor-destroying enzyme and heat-inactivated (30 min, 56°C), tested in 2-fold dilutions starting with an initial dilution of 1∶10, then mixed with 8 HA units of H5N1-VLP and incubated at room temperature. After 1 h, a 0.75% suspension of guinea pig red blood cells was added and hemagglutination was assessed 2 h later by eye. HI titer is expressed as the reciprocal of the highest dilution that showed 50% inhibition of hemagglutination. All samples were tested in triplicate.

ELISA plates (Nunc) were coated with indicated H5 glycoprotein, VLPs, or virus at 4°C overnight and blocked with 1% casein (Blocker Casein, Pierce, Rockford, IL) in PBS. ELISA plates were then incubated with serum samples of indicated dilution for 1 h at 37°C, traced with HRP-conjugated secondary Ab, and developed with TMB substrate (Pierce). They were washed with PBST five times between each step of ELISA. Finally, for the ELISA readout, the absorbance of each well at 450 nm wavelength (*A*_450_) was measured using a microplate reader (Power Wave XS, Bio-Tek).

### Vaccination and viral challenge

Female BALB*/c* mice (6 weeks old) were purchased from the National Laboratory Animal Center, and randomly assigned to receive two doses of vaccine 21 days apart. Vaccines of 0.3 µg, 1.5 µg, 2.5 µg, or 10 µg H5N1-VLPs comprised of either 0.054 µg, 0.27 µg, 0.45 µg, or 1.8 µg of H5 glycoprotein, and whole virus vaccine were grown in chicken embryo, inactivated by formalin and applied at 2.5 µg or 10 µg doses. Vaccines or PBS (as mock control) were given by intramuscular injection into the quadriceps. Blood was collected from mice via the retro-orbital sinus, transferred to a tube containing a serum separator and clot activator, and allowed to clot at room temperature. Sera were removed after centrifugation and stocked at −80°C. The immunized mice were challenged intranasally with a recombinant H5N1 virus, NIBRG-14, with a lethal dose (100-fold lethal dose to 50% of mice) as performed previously [48]. The mice were monitored daily for 14 days after the challenge for survival and morbidity (i.e. weight loss, inactivity and body temperature). All animal experiments were evaluated and approved by the Institutional Animal Care and Use Committee of Academia Sinica.
